# Food contamination and cardiovascular disease: a narrative review

**DOI:** 10.1007/s11739-024-03610-x

**Published:** 2024-05-14

**Authors:** Gerardo Mancuso, Francesco Violi, Cristina Nocella

**Affiliations:** 1 Internal Medicine Unit, Department of Medicine and Medical Specialties, Lamezia Terme Hospital, 88046 Lamezia Terme, Italy; 2 Department of Clinical Internal, Anesthesiology and Cardiovascular Sciences, Sapienza University of Rome, 00161 Rome, Italy

**Keywords:** Cardiovascular disease, Food processing, Contaminants, Environmental factors

## Abstract

Cardiovascular disease is a significant cause of morbidity and mortality among non-communicable diseases worldwide. Evidence shows that a healthy dietary pattern positively influences many risk factors of cardiometabolic health, stroke, and heart disease, supported by the effectiveness of healthy diet and lifestyles for the prevention of CVD. High quality and safety of foods are prerequisites to ensuring food security and beneficial effects. Contaminants can be present in foods mainly because of contamination from environmental sources (water, air, or soil pollution), or artificially introduced by the human. Moreover, the cross-contamination or formation during food processing, food packaging, presence or contamination by natural toxins, or use of unapproved food additives and adulterants. Numerous studies reported the association between food contaminants and cardiovascular risk by demonstrating that (1) the cross-contamination or artificial sweeteners, additives, and adulterants in food processing can be the cause of the risk for major adverse cardiovascular events and (2) environmental factors, such as heavy metals and chemical products can be also significant contributors to food contamination with a negative impact on cardiovascular systems. Furthermore, oxidative stress can be a common mechanism that mediates food contamination-associated CVDs as substantiated by studies showing impaired oxidative stress biomarkers after exposure to food contaminants.This narrative review summarizes the data suggesting how food contaminants may elicit artery injury and proposing oxidative stress as a mediator of cardiovascular damage.

## Introduction

Cardiovascular disease is the key complication of atherosclerosis and the main cause of mortality and morbidity worldwide [1]. Among the factors emerging as a risk for atherosclerosis, diabetes, hypertension, dyslipidemia, and smoking received robust support by observational and interventional with statins, anti-diabetic, and anti-hypertensive drugs. However, a residual atherosclerotic risk remains thereby identification of further cardiovascular risk is still a major challenge of modern medicine. The relationship with the environment is one of the fundamental determinants of the state of health of the human population. The environment can directly or indirectly affect health by promoting the circulation of pathogens and other biological factors, such as pollen and other allergens, but it can also act through non-biological factors, such as the presence of chemical and physical contaminants.

Diet unequivocally plays a key role in the primary and secondary prevention of cardiovascular disease (CVD) [2]. Among the factors that may contribute to an increase in the risk of cardiovascular disease, food contamination is emerging as a novel mechanism that may negatively influence atherosclerotic process and its vascular complications.

The objective of the current narrative review was to briefly describe how food contamination may elicit artery damage by identifying some of food contaminants that can affect cardiovascular health and the underlying mechanism. Specifically, we described the role of oxidative stress as a mechanism linking food contamination to cardiovascular diseases.

## Definition of food contaminants

According to the World Health Organization (WHO), food contamination represents a global challenge [3], as the higher concentration of contaminants could imply serious health risks**.** The safety and quality of foods are influenced by various conditions such as temperature and humidity, primary treatment, condition at capture or harvest, and storage conditions. Moreover, the man-made alteration of the environment can contribute to the input of chemical environmental compounds that, by contaminating soil, water, and air, ultimately impact the food chain.

Therefore, contaminants can be defined as a group of heterogeneous compounds derived from several sources. Food contaminants may result from the different stages of food production, processing, packaging, and transport. They may be also present in food from environmental contamination, that may alter the nutrients predisposing to vascular damage. The most important contaminants present in food and feed include natural toxins such as alkaloids or mycotoxins, environmental contaminants following industrial or agricultural activities and include polychlorinated biphenyls, dioxins, chlorinated pesticides, acrolein, metals, such as arsenic, cadmium, lead, mercury (Table 1).

**Table 1:** Types of food contaminants and foods they are mainly present

Contaminants	Foods
Metals	Fresh meat Bivalve mollusks Fishing products Fresh and frozen vegetables Tubers Legumes Fruit Cocoa Alcoholic beverages Food supplements
Radioactive contaminants	Fresh meat Milk and derivatives Mushrooms
PAHs	Meats Fishing products Vegetables oils Vegetables fats Flour for bread and pizza Cocoa seeds
Nitrates	Fresh vegetables
Polar compounds acrolein	Frying oil
Sulfur dioxide	Alcoholic beverages

*PAHs* polycyclic aromatic hydrocarbons

## Intrinsic food contamination: production, processing, storage, and preparation phases

Food processing can profoundly affect diet quality; however, there is a broad spectrum of food processing, ranging from minimal processing (e.g., frozen, dried vegetables, fruit with no added sugar or additives, pasteurized milk) to ultra-processing (e.g., soda, fast food, industrially produced bread, hot dogs) [4]. Ultra-processed foods (UPFs), the result of repeated industrial processes, are tasty and pleasant on the palate, keep for a long time, are packaged and finished foods to be reheated and ready to be consumed. Ultra-processed foods include not only so-called “junk” foods, but also wide array of foods marketed and perceived as healthy, such as flavored yogurts, low-fat, low-calorie products, breakfast cereals, and nutrient-enriched products, ‘energy’ drinks, and sugared drinks [4].

The recent epidemiological studies suggest that a greater consumption of UPFs is associated with an increased risk of CVDs. In a population-based cohort of 91.891 participants, after an average follow-up of 13.5 years, a high consumption of UPFs was associated with increased risks of overall cardiovascular and heart disease mortality, including heart disease deaths and cerebrovascular deaths [5]. A recent metanalysis of a total of 39 cohort studies, involving more than 60 million participants, showed that a higher consumption of UPFs, up to 1 serving per day, significantly increases the risk of developing cardio-cerebrovascular diseases [6].

### Artificial sweeteners

Processing can alter both macro and micronutrient nutritional characteristics, physical characteristics such as the structure of the food, and chemical characteristics due to the presence of natural or artificial sweeteners, altering the glycemic index of foods with elevation of insulin and insulin resistance.

An example of chemical interference is fructose, some sugar widely used in the preparation of foods, especially drinks, with much lower costs than glucose but with superior organoleptic characteristics such as the almost non-existent property of crystallizing. Our body can metabolize 30–40 g of fructose per day due to the inability of GLUT5 and fructokinase, but the daily intake in industrialized populations has increased considerably up to 120 g. This leads to an increase in insulin resistance and uric acid and all those pathologies related to it such as metabolic syndrome, diabetes mellitus, Nonalcoholic Fatty Liver Disease (NAFLD), factors directly related to an increase in cardiovascular risk.

Artificial sweeteners have been used as sugar alternatives since the 1800s, but their consumption has significantly increased in the recent years. Even if there are no reports by randomized controlled trials investigating if long-term intake of artificial sweeteners results in negative cardiovascular consequences, several other studies associated artificial sweeteners with an increased risk of metabolic syndrome, hypertension, insulin resistance and dyslipidemia [7]. Artificial sweeteners may contribute to cardiovascular disease 1) by the alteration of gut microbiota, disrupting the balance of gut bacteria species [8]; 2) favoring the acceleration of atherosclerosis via impairment of function and structure of high-density lipoprotein (HDL) and its major protein constituent, ApoA-I [9]; 3) by the regulation of cardiac electrophysiology by altering heart’s electrical conduction system [10].

Among artificial sweeteners widely used in ultra-processed foods and especially in artificially sweetened beverages, some snacks, and low-calorie ready-to-go meals or dairy products, aspartame, acesulfame potassium, and sucralose were found associated with detrimental effects on cardiovascular health [11]. The analysis of the consumption of artificial sweeteners from all dietary sources in the prospective NutriNet-Santé cohort revealed that aspartame intake was associated with an increased risk of cerebrovascular events whereas acesulfame potassium and sucralose were associated with increased coronary heart disease risk [11].

A recent study by Witkowski and colleagues showed that circulating levels of erythritol were associated with incident risk for major adverse cardiovascular events [12]. Moreover, erythritol enhances platelet responsiveness, and in vivo thrombosis formation in human blood and the carotid arteries of mice [12].

### Food additives and contaminants

Food additives and contaminants newly formed during processing may also play a role in cardiovascular risk. Additives contained into UPFs with adverse cardiometabolic effects include for example glutamates [13, 14], emulsifiers [15, 16], and sulfites [17]. In chronic alcoholic (30% ethanol/100 g body weight) and normal adult male mice, the oral ingestion of monosodium glutamate (4 mg/g body weight and above) increased lipid peroxidation and decreased the levels of endogenous antioxidants [14]. In adults with Type 2 Diabetes, higher intake of glutamate was associated with a higher risk of CVD incidence, CVD mortality, and total mortality [13]. Emulsifiers are detergent-like molecules that are incorporated into many processed foods in order to improve texture and extend shelf life [18]. In mice, relatively low concentrations of two commonly used emulsifiers, carboxymethylcellulose and polysorbate-80, induces microbial dysbiosis and low-grade inflammation promoting metabolic syndrome [15]. In a prospective cohort study including 95.442 adults without prevalent CVDs, a positive association between higher intake of food additive emulsifiers (celluloses, carboxymethylcellulose, monoglycerides and diglycerides of fatty acids) and the risk of CVDs and coronary heart disease was found [16].

During food processing, heat treatments produce neo-formed toxic compound such as acrylamide, a by-products that can be formed during the Maillard reaction [19]. Several studies suggest that acrylamides in UPFs is associated with cardiovascular disease [20–23]. The baseline examination of 8.290 adults from National Health and Nutrition Examination Survey (NHANES) 2003–2006 with self-reported diagnosis of CVDs, showed that hemoglobin adducts of acrylamide, biomarkers of internal exposure to acrylamide, were significantly associated with CVDs in smokers [23]. Moreover, acrylamide was associated with platelet activation and suppression of circulating angiogenic cell levels, as well as increased risks of CVDs [24].

Other mechanisms underlying the correlation between ultra-processed food intake and CVDs disease include the presence of bisphenol A (BPA) in the materials that make up ultra-processed food containers. BPA, which is very similar in structure to 17 beta estradiol, promotes insulin resistance, oxidative stress, inflammation, adipogenesis, pancreatic beta cell dysfunction by binding to estrogen-related receptors [25].

All these compounds and characteristics of UPFs synergize to influence cardiovascular health involving complex mechanisms which are not yet fully understood. However, key potential biological mechanisms underlying the association between UPFs and CVD include the dyslipidemia, changes in the intestinal microbiota, body composition with an increase in fat mass, promotion of inflammation and oxidative stress phenomena, insulin resistance and blood pressure [26].

Nitrite and nitrate salts are commonly used to season meat and other perishable products, such as cheese. They are added to foods to preserve them and help to hinder the growth of harmful microorganisms. They are naturally present in vegetables, especially leafy ones, such as lettuce and spinach and in water as they are used as fertilizers. The Acceptable Daily Intakes (ADI) for nitrite, established by the European Commission's Scientific Committee for Food (SCF) in 1997 and the Joint FAO-WHO Committee on Food Additives (JECFA) in 2002, are respectively 0.06 and 0.07 mg per kilogram of body weight per day (mg/kg bw/day). In the case of nitrate, on the other hand, both institutions set the ADI at 3.7 mg/kg bw/day.

When taken with the diet they are rapidly absorbed by the body and excreted in the urine. Nitrates can also survive passage through the stomach and enter the circulatory system. A variety of highly bioactive reactive nitric oxide species are formed in the acidic environment of the stomach or in blood and tissue. These may be involved in the generation of nitrosamines of toxicological importance when nitrites combine with secondary amines present in the stomach resulting in an increased risk of gastro-intestinal cancer [27]. The presence of antioxidants in the diet inhibits the generation of nitrosamines. However, there are also benefits of dietary intake of nitrates and nitrites, which have been demonstrated in many studies. The positive effect of nitrates and nitrites is linked to the fact that they are exogenous donors of NO, which has a potentially beneficial role in physiology and therapy [28]. The widely considered and described benefit of taking nitrates and nitrites is its positive effect on the cardiovascular system. The impact of nitrate and nitrite intake on endothelial function and blood pressure is extensively studied [29–31].

N-nitrosamines (N-NA) in food represent harmful elements for health and public health when the quantities are high. This category includes the 10 food carcinogens (TCNA), namely NDMA, NMEA, NDEA, NDPA, NDBA, NMA, NSAR, NMOR, NPIP and NPYR. N-NAs are genotoxic and induce liver tumors in experimental animals and the available in vivo data are limited to detect their potency. The tolerated limit is 10 μg/kg of body weight per day beyond which the incidence of rat liver tumors is relevant, especially from NDEA. The dietary exposure of TCNAs was evaluated for cooked meat and fish. Exposures ranged from 0 to 208.9 ng/kg body weight per day. However, meat and meat products are the main food category contributing to exposure to TCNA. Only one experimental study was conducted about the role of N-nitrosamines as risk factors for the incidence of cardiovascular disease. The treatment of rats with 0.2 mg/kg body weight of several TCNAs for two weeks altered the lipid profile by increasing cholesterol and LDL levels and decreasing HDL [32]. Moreover, TCNAs treatment increased levels of free radicals and decreased the activity of antioxidant enzymes such as glutathione levels, and glutathione reductase [32].

## Extrinsic food contamination: environmental factors

Air pollution is an environmental risk factor for mortality. Approximately 2/3 of deaths attributable to air pollution occur from cardiovascular causes (ischemic heart disease and cerebrovascular disease) [33]. Air pollution encompasses a wide range of substances derived from many different sources and chemical reactions within the atmosphere that enter the food chain. Food is the fundamental element through which pollutants enter the food cycle.

Although hypertension, obesity, and metabolic disorders such as diabetes and dyslipidemia are major risk factors for cardiovascular disease, environmental factors result in additional synergistic or additive effects to these well-established risk factors.

In 1999 the Agency for Toxic Substances (ATSDR) of the United States of America published a list of 275 organic and inorganic substances dangerous for humans and the environment. In the list of the 20 most dangerous substances there were 5 heavy metals. Arsenic, lead, mercury, cadmium, and chromium occupied the 1st, 2nd, 3rd, 7th, and 16th positions, respectively.

### Heavy metals

Heavy metals are environmental contaminants that pose an increasing ecological and global public health concern. According to the World Health Organization, 60–0% of all chronic and acute diseases can be traced back to heavy metal poisoning. Chronic exposure to low and low-moderate levels of metals such as arsenic (As), lead (Pb), and cadmium (Cd^2+^), constitutes a significant risk factor for CVD, including ischemic heart disease, stroke, and peripheral artery diseases (PAD) [34].

#### Arsenic

Arsenic is a potent toxic and carcinogenic metalloid that can be assumed because it is widespread in water, soil, and some foods [35]. From the soil, arsenic passes into plants which tend to accumulate in their tissues, therefore the level of Arsenic in crops will depend on the level of this metal in the soil.

The association between arsenic and cardiovascular diseases is relatively well-established in adults as described by several studies [36–38]. Indeed, the revision of epidemiologic studies about high-chronic arsenic exposure showed a causal association between high exposure to arsenic in drinking water (> 50 μg/L) and cardiovascular disease, coronary heart disease, stroke, and peripheral arterial disease [36]. A very recent study showed a significant association between elevated total arsenic levels and greater carotid intima-media thickness in children [39]. Moreover, a cohort study investigated the relationship between heavy metals in high-consumption foods such as rice, bread, and vegetables, and cardiovascular disease [40]. By the measurement of heavy metals in the urine, the authors found that, among several heavy metals that enter the human body through contaminated food, arsenic levels in vegetables and rice are more than the standard limitation value, and it is associated with CVDs. Finally, cardiovascular patients showing higher arsenic deposits in their fingers and higher cardiac tissue injury scores, showed shorter telomere length, decreased mitochondrial DNA copy number, and increased mitochondrial DNA (mtDNA) deletion [41]. These changes can explain increased cardiovascular risk as short leukocyte telomere lengths and mtDNA alterations in terms of length and location may participate in atherogenesis [42, 43].

#### Lead

The general population is exposed to lead which is distributed in the air and food in roughly equal proportions. There are a variety of lead sources including old paint, tobacco products and secondhand smoke, contaminated foods and drinking water that have caused a significant increase in this metal in the environment. Among Chinese adolescents and adults, the blood lead levels of 5 μg/dL play an important role in the disease burden of CVDs, including stroke ischemic, hypertensive, and rheumatic heart diseases. Dietary lead intake was a major contributor to the disease burden (68.1%) [44]. Moreover, children are susceptible to lead exposure due to high gastrointestinal absorption and permeability of the blood–brain barrier [45].

Cadmium compounds are currently mainly used in nickel–cadmium rechargeable batteries. Cadmium emissions have increased dramatically over the last century as batteries are rarely recycled and often thrown away in household waste, contaminating soil and entering food production cycles. Cadmium can cause kidney damage [46] but probably also effects bones by reducing their hardness and causing fractures [47]. A recent review examined several epidemiological studies on cadmium and the risk of atherosclerotic cardiovascular disease (ASCVD) [48]. The results showed that cadmium increased the risk of ASCVD and asymptomatic atherosclerosis in the carotid and coronary arteries above suggested exposure level (> 0.5 μg/L for blood cadmium or > 0.5 μg/g creatinine for urinary cadmium). Interestingly, the analysis of potential underlying mechanisms of cadmium-related cardiovascular risk, showed that cadmium accumulates in arterial walls favoring atherosclerotic process. Indeed, cadmium exposure can contribute to hyperlipidemia, increase LDL oxidation, reduce NO bioavailability and endothelial dysfunction via oxidative stress and inflammation [48].

One such chemical found as a contaminant in food, air and water is acrolein (2-propenal, CH2 = CHCHO), a colorless volatile liquid in pure form with an unpleasant odor.

#### Tungsten

Tungsten is a unique metal element classified by US Environmental Protection Agency as an emerging contaminant. The general population may be exposed to Tungsten through inhalation of air, drinking water, and food consumption. Despite its large use and several data indicated that tungsten compounds could even enter the food chain, the potential environmental effects of tungsten released in the environment are unknown and few data are available to make an environmental risk assessment [49].

A recent analysis of participants in the National Health and Nutrition Examination Survey (NHANES) showed that Tungsten enhances the risk of CVDs, especially congestive heart failure, coronary heart disease, and angina pectoris (AP) [50]. The Monocyte-to-HDL-C ratio, monocyte count, high-density lipoprotein cholesterol and white blood cell count played a mediating role between Tungsten and CVDs [50].

#### Mercury

Fish is the major source of mercury deposits in the human food chain. Shark, swordfish and tuna, and fish such as pike, walleye, and bass, taken from polluted freshwater should be avoided.

Mercury, once absorbed, binds to the sulfhydryl groups of erythrocytes in the blood, crosses the blood–brain barrier, even if partially, through the cysteine transporter and accumulates in the brain. Mercury, which has a half-life of 42 days, accumulates in the placenta, in the fetal tissues and in the amniotic fluid, in the liver in the periportal portion and in smaller quantities in the epithelial tissues, in the kidneys, in the choroidal plexus and in the testicles. Mercury interferes with DNA transcription and protein synthesis causing cell damage. There is evidence of the cause of some diseases such as Alzheimer, impaired embryogenesis, Minamata disease [51].

A Norwegian study has shown that levels of some metals in fish are below the levels allowed under the old legislation, however the same levels can be harmful to children, who could run the risk of exceeding the tolerable weekly intake of mercury when they eat products with the highest levels of mercury [52]. The presence of these metals in fish is a health problem. Estimated weekly intakes (EWI) for cadmium, lead, and mercury were evaluated and compared with the tolerable standard weekly intakes (TWI) for cadmium for blue mussels, soft clams, European squid, veined squid, shrimp rockfish, red mullet, sea bass, sea bream, cod, European hake, Atlantic bluefin tuna and swordfish. But if for all metals, the weekly amount consumed by Italian consumers was below those allowed for cadmium, this was not the case. In 2021 the levels of metals have been reviewed by the European Community and all these metals are all above the permitted levels [53]. In a European study, the levels of cadmium, lead, mercury, and arsenic were measured in 39 species of fruit and fruit processed for the food industry by analyzing the products with optical emission spectrometry (ICP-OES). The study highlighted how 23.9% of the 377 samples examined showed an increase in the maximum levels of metals compared to the values established by the European Commission [53]. Exceeding the levels of these metals shows that attention needs to be paid to food compliance. Increased serum concentrations of Cu, Se and Cr may be associated with an increased incidence of cardiovascular disease.

### Chemical compounds

#### Acrolein

One such chemical found as a contaminant in food, air and water is acrolein (2-propenal, CH2 = CHCHO), a colorless volatile liquid in pure form with an unpleasant odor.

Acrolein is an unsaturated aldehyde that is formed during the combustion of fuels, cigarettes, wood, plastic, and during the cooking or frying of foods with fats or oils. It is a product of polyamine oxidation and lipid peroxidation which causes DNA damage, activates inflammation processes, ROS formation, protein adduction, mitochondrial dysfunction, and damage to the retina [54].

Acrolein is also continuously generated by biological systems subjected to oxidative stress. Acrolein is a ubiquitous environmental pollutant that can cause adverse effects in the central and peripheral nervous system, respiratory tract, and cardiovascular system. At the cellular level, acrolein causes toxic effects through potentiation of reactive oxygen species (ROS), DNA, and protein adducts, by induction of endoplasmic reticulum (ER) stress and immune dysfunction, and by cell membrane damage and mitochondrial disruption [55]. Moreover, plasma levels of acrolein protein conjugate were reported to be related to the development of carotid atherosclerosis [56].

**Fig. 1:**
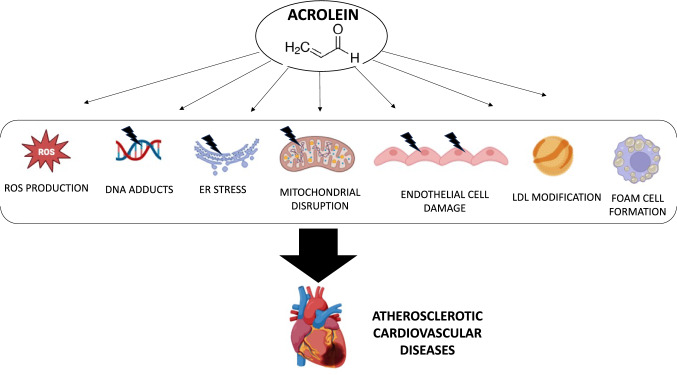
Acrolein-mediated cardiovascular injury**.**At the cellular level, acrolein exerts several toxic effects. Acrolein disrupts redox homeostasis and contributes to the generation of ROS, DNA and proteins adducts, induces *ER* endoplasmic reticulum stress and immune dysfunction, cell membrane damage and mitochondrial disruption. Moreover, acrolein favors endothelial cell damage, LDL modification, and facilitates foam cell formation, contributing to the initiation and developmental stages of atherosclerosis

These findings are supported by other evidence showing that acrolein contributes to the initiation and developmental stages of atherosclerosis, including endothelial cell damage, LDL modification, enhancement of the inflammatory response, and expression of scavenger receptors on the surface of macrophages that facilitate foam cell formation [57]. More importantly, some studies demonstrate the role of acrolein exposure in the development of dyslipidemia, platelet activation, platelet − leukocyte aggregates, and thrombosis [24, 58], the well-known risk factors for heart and brain complications (Fig. 1).

The cardiovascular system is sensitive to the toxic effects of acrolein because it can generate stress from oxygen-free radicals, decrease the phosphorylation of endothelial nitric oxide synthase and the formation of nitric oxide, carry out cytoplasmic and nuclear reactions with myocytes and endothelial cells vascular and cause vasoconstriction. Thus, chronic exposure to acrolein can cause myocyte dysfunction, myocyte necrosis, and apoptosis, and ultimately lead to cardiomyopathy and heart failure [59]. It has been demonstrated that after cranial trauma, acrolein increases, generating a procoagulant effect. Acrolein produced in the perilesional cerebral cortex may contribute to an early hypercoagulable state by interfering with the metabolism of von Willebrand factor (VWF) [60]. Acrolein may also play a role in myocardial infarction. When acrolein is given with isoprenaline (ISO) it produces a myocardial infarction, but when acrolein is made less active when given with hydroxytyrosol and oleuropein the myocardial infarction induced by isoprenaline is less intense [61]. Acrolein is an irritant to the endothelium. Even that produced by the combustion of internal combustion engines enters the food cycle when the crops are near roads with heavy traffic. The area of endothelium particularly sensitive to acrolein is the carotid body which controls changes in blood pressure and oxygen and carbon dioxide levels and interferes with downstream cardiac function control. Rats exposed to acrolein showed an increase in blood pressure and a reduction in myocardial contractility [62].

#### Dioxins and polychlorinated biphenyls (PCBs)

Dioxins and polychlorinated biphenyls (PCBs) are organic environmental persistent chemicals contaminants with similar toxicological properties, classified as persistent organic pollutants (POPs) [63]. Because these compounds are fat soluble and accumulate in the marine food chain, they could have adverse health effects including CVDs together with other pathologies such as diabetes, cancer, or altered immunologic response and altered metabolism [63].

POPs exposure remains of concern to the international community since they still accumulate in the food chain resulting in low but continuous human exposure. Dioxins and polychlorinated biphenyls have received the most attention regarding their concentration in fish. However, most fish on the commercial market are regularly monitored for their dioxin levels and self-caught fish at presumed non-contaminated sites do not represent a major health risk [64]. Indeed the link between fish consumption and the reduction of cardiovascular disease risk was classified as convincing by the Joint WHO/FAO Expert Committee [65].

There are several data about the relationship between dietary exposure to dioxins and PCBs and cardiovascular health in the general population, although some results are discordant. A recent, large cohort study including more than 400.000 adults showed that the exposure to dioxins, or PCBs was not associated with cardiovascular mortality [66]. However, other studies indicated that dietary exposure to PCBs has been associated with an increased risk of myocardial infarction [67], hemorrhagic stroke [68], higher risk of developing hypertension [69] and subclinical coronary atherosclerosis [70]. Conversely, no association was found between dietary dioxins and coronary atherosclerosis [70].

### Oxidative stress as a mechanism of food-related cardiovascular diseases

**Fig. 2:**
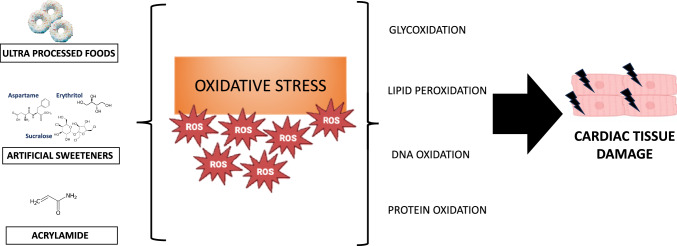
Oxidative stress-mediated tissue damage Oxidative stress is one of the mechanisms that determines tissue damage. Some dietary habits and factors influence oxidative stress, and lead to oxidative lesions of DNA, proteins, lipids, and sugars. In cardiomyocytes, the oxidative modification of major cellular macromolecules induces modifications in subcellular organelles such as sarcolemma, mitochondria, sarcoplasmic reticulum, and nucleus leading to contractile dysfunction and cardiomyocytes hypertrophy

Oxidative stress is one of the mechanisms that determines tissue damage. Oxidative stress is defined as a condition that reflects an imbalance between the ROS production and antioxidant’s ability to readily detoxify them. Oxidative stress is closely related to inflammation [71], and this interrelation represents a probable means by which the effects of contaminants can be amplified to produce pathophysiological effects in multiple tissue, principally in cardiac tissue (Fig. 2).

Several types of food contaminants can affect oxidative balance. High UPF consumers among old adults with metabolic syndrome displayed significant changes in oxidative status. ROS production in neutrophils was increased whereas catalase and superoxide dismutase activities were lower in high UPF consumers. In addition, biomarkers of inflammation, tumor necrosis factor α (TNFα), interleukin (IL)-6, IL-15, and leptin levels were higher in participants with high intake of UPF suggesting significant changes in pro-oxidant and inflammatory profile associated with high UPF [72]. Sweeteners also impair oxidant–antioxidant balance. Urinary concentration of acesulfame and saccharin showed a significant positive interaction with 8-OHdG and malondialdehyde (MDA) [73], that are both good oxidative damage indicators for effect of ROS on DNA and lipid molecules, respectively [74]. In vitro, the sucralose concentration, that could plausibly be observed in the circulatory system of high non-nutritive sweeteners consumers, significantly increases ROS accumulation [75]. In addition, aspartame, or aspartame metabolites, such as aspartic acid, phenylalanine, and methanol generated after digestion of aspartame in the human intestinal tract, increased oxidative stress and mitochondrial damage in vitro [76].

Finally, acrylamide exposure significantly increased ROS production, MDA levels and glutathione (GSH) consumption in association with increased levels of pro-inflammatory cytokines tumor necrosis factor-α (TNF-α) and interleukin 6 (IL-6) [77].

## Limitations

The current narrative review does have limitations. Our search strategy may not have identified all studies reporting the relationship between food contaminants and CVDs. Moreover, the subjective nature of the determination of which studies to include, the interpretation of the results, and the conclusions drawn represent other limitations of this study. Although this was not a systematic review of current literature with rigorous data extraction, the studies we reviewed provided an overview of food contaminants-related cardiovascular risk. However, some of these studies reported discordant results. Moreover, most of the evidence is derived by population association studies or by vitro or animal studies, and a causal relationship cannot be established. Therefore, further well-controlled, long-term human studies are warranted to provide robust conclusions.

## Conclusions

In conclusion, food contamination can pose a serious health hazard. Contamination can come from different situations. Food processing can profoundly influence diet quality. Specifically, ultra-processed foods, defined as ready-to-eat or ready-to-heat food that has undergone intense industrial physical, chemical, or biological processes [78], may affect health outcomes by influencing lipid concentrations, glycemic response, and the gut microbiota composition and function [26], predisposing to CVD. Moreover, extensive literature and epidemiological studies showed that environmental pollutants, in particular heavy metals such as lead, cadmium, and arsenic, are significant contributors to CVD. Therefore, public health measures such as an increased controls of soil, water, and prepackaged foods, are necessary to ensure better food quality and therefore diminish the burden of CVD attributable to food contamination.

## Data Availability

Data sharing is not applicable to this article as no new data were created or analyzed in this study.
